# Effects of acupuncture on vascular dementia (VD) animal models: a systematic review and meta-analysis

**DOI:** 10.1186/s12906-018-2345-z

**Published:** 2018-11-13

**Authors:** Ze-Yu Zhang, Zhe Liu, Hui-Hui Deng, Qin Chen

**Affiliations:** 1 Department of Traditional Chinese Medicine, Third Clinical Medical College of Zhejiang Chinese Medical University, Hangzhou, Zhejiang China; 2 Department of of Neurobiology and Acupuncture Research, Third Clinical College of Zhejiang Chinese Medical University, Hangzhou, Zhejiang China; 3 Department of Acupuncture, Zhejiang Chinese Medical University Affiliated Third Hospital, Hangzhou, Zhejiang China

**Keywords:** Acupuncture, Vascular dementia, Systematic review, Meta-analysis

## Abstract

**Background:**

Vascular dementia is the second most common type of dementia that causes cognitive dysfunction. Acupuncture, an ancient therapy, has been mentioned for the treatment of vascular dementia in previous studies. This study aimed to evaluate the effects of acupuncture in animal models of vascular dementia.

**Methods:**

Experimental animal studies of treating vascular dementia with acupuncture were gathered from Embase, PubMed and Ovid Medline (R) from the dates of the databases’ creation to December 2016. We adopted the CAMARADES 10-item checklist to evaluate the quality of the included studies. The Morris water maze test was considered as an outcome measure. The software Stata12.0 was used for the meta-analysis. Heterogeneity was examined using I^2^ statistics, and we conducted subgroup analyses to determine the causes of heterogeneity for escape latency and duration in original platform.

**Results:**

Sixteen studies involving 363 animals met the inclusion criteria. The included studies scored between 4 and 8 points, and the mean was 5.44. The results of the meta-analysis indicated remarkable differences with acupuncture on increasing the duration in the former platform quadrant both in EO models (SMD = 1.56, 95% CI: 1.02 ~ 2.11; *p* < 0.00001) and 2-VO models (SMD 4.29, 95% CI 3.23 ~ 5.35; *p* < 0.00001) compared with the control groups.

**Conclusions:**

Acupuncture may be effective in improving cognitive function in vascular dementia animal models. The mechanisms of acupuncture for vascular dementia are multiple such as anti-apoptosis, antioxidative stress reaction, and metabolism enhancing of glucose and oxygen.

**Electronic supplementary material:**

The online version of this article (10.1186/s12906-018-2345-z) contains supplementary material, which is available to authorized users.

## Background

Vascular dementia (VD) is a heterogeneous group of brain disorders of which the cognitive impairment can be ascribed to cerebrovascular pathologies; more than 20% cases of dementia are vascular dementia, making it the second most prevalent form of dementia, second only to Alzheimer’s disease (AD) [1]. Advanced age, diabetes, hypertension, smoking and atrial fibrillation are all risk factors for vascular dementia [2]. Level of education, which is considered to be an effective alternative indicator of cognitive reserves, has a significant association with the expression of VD in that higher education appears to be associated with fewer cognitive deficits [3, 4]. As the populations of North America and European countries age, the risk of VD in these regions approximately doubles every 5.3 years. However, the current study suggests that effective therapy for vascular dementia has proven to be more difficult than for Alzheimer’s disease. While memantine and cholinesterase inhibitor drugs have been proven to be significantly effective in treating Alzheimer’s disease and are therefore labeled for this indication, they are not recommended for use in the treatment of vascular dementia by either regulatory bodies or guideline groups due to their overall low effectiveness and possible side effects [5–7].

Acupuncture, an economical type of traditional Chinese therapy with minimal side effects, has been used for many diseases in Asian countries for thousands of years and is widely used for rehabilitation after stroke [8]. Usually after a stroke, 15–30% of subjects will develop vascular dementia within three months, and delayed dementia will develop in the long term due to recurrent stroke in 20–25% of subjects [9]. In recent years, more and more studies, and especially animal experiment studies, have been published to illustrate the effectiveness of acupuncture for vascular dementia.

Until now, no systematic meta-analysis has been published to analyze the effects of acupuncture on enhancing cognitive function in vascular dementia animal models. A systematic review of animal experiments can be beneficial for future experimental designs and provide a basis for clinical studies. Additionally, it can provide valuable directions for further research. It is for these reasons that we conducted this systematic review and meta-analysis.

## Methods

This systematic review and meta-analysis complied with standard guidelines (See Additional file 1).

### Search strategy

The following electronic databases were searched from database inception to December 2016: Embase, PubMed and Ovid Medline (R), and the following keywords were used: acupuncture, electroacupuncture, acupoint, vascular dementia, multi-infarct dementia and multiinfarct dementia. The search language was limited to English, and we also tried to collect records from other sources. The detailed search strategies are shown in Additional file 2.

### Inclusion criteria

1. Subjects: Animal models of vascular dementia were included.
2. Intervention: Only manual acupuncture and electroacupuncture (EA) were included.
3. Outcome: The Morris water maze test was used to evaluate cognitive functions in animal models.
4. Language: Only articles published in English were included.
5. No publication date limit was set, and the search was conducted in December 2016.

### Exclusion criteria

1. Duplicate articles;
2. Studies that have no control group;
3. Auricular acupuncture, laser acupuncture and other acupuncture techniques;
4. Acupuncture therapy combined with the use of traditional Chinese medicines or Western drugs;
5. Studies aiming to compare different acupuncture techniques;
6. Studies that only compared acupuncture with traditional Chinese medicines.

### Study selection and data extraction

One reviewer (ZYZ) generated the search strategy, searched the databases, and made a list of all the records. Two evaluators (HHD, QC) independently evaluated the articles based on the inclusion and exclusion criteria. Disagreements were solved together through discussions (ZYZ, HHD, QC). Two reviewers (HHD, QC) independently extracted the data.

The following data were extracted: publication year, the last name of the first author, model of vascular dementia, weight range of the included animals, number of animals included, method of treatment with timing and duration in the acupuncture and control groups, the assessment of trials, and the results of each article (positive or negative). The final outcomes were extracted if several outcomes were presented. When the outcome data were only shown graphically, we attempted to contact the authors to obtain the detailed data; if we received no response to our request, we used GetData software to measure the data. Differences were solved together through discussions (HHD, QC, ZYZ).

### Risk of bias assessment

We evaluated methodological quality against an ten-item checklist [10]: (1) peer-reviewed journal; (2) temperature control; (3) animals were randomly allocated; (4) blind established model; (5) blinded outcome assessment; (6) anesthetics used without marked intrinsic neuroprotective properties; (7) animal model (diabetic, advanced age or hypertensive); (8) calculation of sample size; (9) statement of compliance with animal welfare regulations; (10) possible conflicts of interest.

The quality of each study was evaluated by a score from zero to ten. Two evaluators (HHD, QC) independently extracted the data and assessed the quality of each study. Disagreements were resolved through discussion (HHD, QC, ZYZ).

### Statistical analysis

For statistical analysis, we used the statistical software package Stata version 12.0. The data of the Morris water maze test, such as escape latency, time in the quadrant in which the former platform was located, and frequency of crossing through the former platform were considered continuous data, and we therefore calculated standard mean differences (SMD) with confidence intervals (CIs) established at 95%. Heterogeneity in the studies was examined using I^2^ statistics. If the I^2^ statistic was higher than 50%, we considered significant heterogeneity to be present, and we used a random effects model. Otherwise, we used a fixed effect model. When significant heterogeneity existed, the subgroup analysis would be conducted based on animal species, acupuncture methods and modeling methods. We used Egger’s test and Begg’s test to assess publication bias.

## Results

**Fig. 1:**
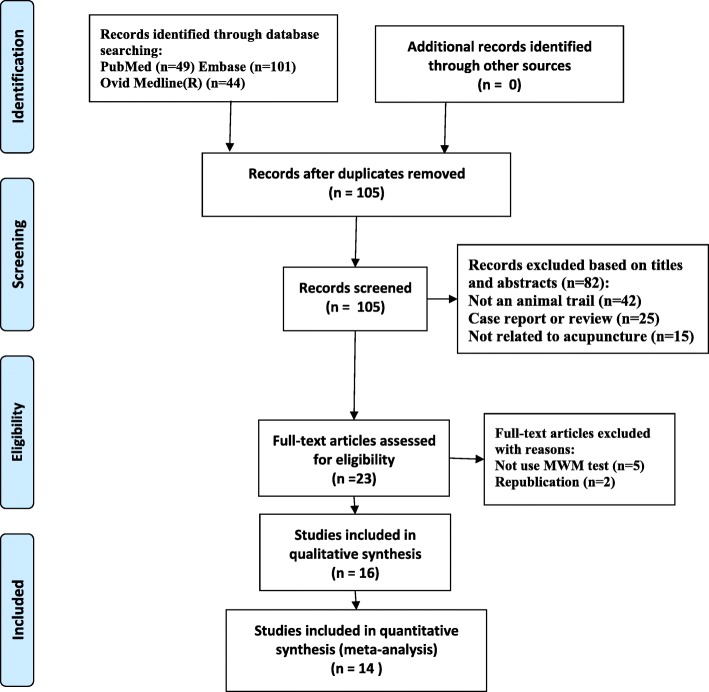
Flow diagram of the study selection process. MWM:Morris water maze

We identified 194 possibly relevant studies in the initial search. After duplicates had been removed, we screened the titles and the abstracts of 105 remaining records, and 82 records were excluded. For further screening, 23 remaining articles were downloaded. Eventually, 16 studies met the inclusion criteria [11–26]. The selection process flow diagram is shown in Fig. 1 [27].

### Study characteristics

The 16 studies included involved 363 rats, 174 of which were in an acupuncture group and 189 of which were in a control group. All the studies stated the weight range of the rats, which ranged from 180 to 490 g. A total of 7 of the 16 studies mentioned the age of the animals [11, 14, 16–18, 23, 25], which ranged from 2 months old to 12 months old. Sixteen of the included studies used different subtests of the Morris water maze test; all the studies met the inclusion criteria by using escape latency as outcome data, while two studies used swimming speed [24, 26], five studies used duration in the quadrant of the former platform position [12, 13, 21, 24, 26], one used swimming distance [19], and three used frequency of crossing former platform [12, 15, 16]. Two species of rats were used in the 16 studies: seven studies used Sprague–Dawley (SD) rats [11, 13, 15, 17–19, 25], and nine studies used Wistar rats [12, 14, 16, 20–24, 26]. The basic characteristics of the studies are listed in Table 1 [28].

**Table 1:** Data of 16 included studies

Trial	Species(Na/Nc)	Age(month)	Weigh(g)	Model	Acupuncture(acupoints)	Control intervention	Outcome assessment	Result
Wang 2004 [11]	SD rats (14/13) SD rats (14/13)	2~ 3	200–250	4-VO	EA,20 min/d for 15d, 150HZ, 2 mA, continuous Waveform (GV14, GV20)	No treatment Nimodipine	Escape latency Escape latency	*P* < 0.01 *P* > 0.05
Yu 2005 [12]	Wistar rats (15/14)	NR	340 ± 40	EO	manual acupuncture, 30 s/d for 21 d (CV6, CV12, CV17, SP10, ST36)	Placebo-acupuncture	Escape latency Duration in former platform position Frequency crossing former platform	*P* < 0.05 *P* < 0.05 *P* < 0.05
Shao 2008 [13]	SD rats (9/8) SD rats (9/8)	NR	180–220	4-VO	EA,20 min/d for 15 d, 150 HZ, 1–2 mA, Continuous Waveform (BL17, BL20, BL23, GV20)	No treatment Nimodipine	Escape latency Escape latency	*P* < 0.01 *P* > 0.05
Wang 2009 [14]	Wistar rats(11/11)	10	300 ± 40	EO	manual acupuncture, 30 s/d for 21 d (CV6, CV12, CV17, SP10, ST36)	Placebo-acupuncture	Escape latency Duration in former platform position	*P* < 0.05 *P* < 0.01
Wei 2011 [15]	SD rats(10/10)	NR	200–250	2-VO	EA, 20 min/d for 10 d, 50 HZ,1.0 mA, continuous Waveform (GV14, GV20)	No treatment Nimodipine	Escape latency Frequency crossing former platform Escape latency Frequency crossing former platform	*P* < 0.01 *P* < 0.01 *P* > 0.01 *P* > 0.01
Zhao 2011 [16]	Wistar rats(10/10)	4	240 ± 20	EO	manual acupuncture, 30 s/d for 21 d(CV6, CV12, CV17, SP10, ST36)	Placebo-acupuncture	Escape latency Swimming Speed	*P* < 0.01 *P* > 0.05
Zhu 2011 [17]	SD rats(11/12)	9	460 ± 30	2-VO	EA, 20 min/d for 30 d,4HZ, 2.0 mA, continuous waveform (BL23, GV14, GV20)	No treatment	Escape latency	*P* < 0.01
Zhu 2012 [18]	SD rats(12/10)	12	400 ± 30	2-VO	EA, 20 min/d for 30 d,4 HZ, 2.0 mA, continuous waveform (BL23, GV14, GV20)	No treatment	Escape latency	*P* < 0.05
Zhu 2013 [19]	SD rats(6/6)	NR	432 ± 30	2-VO	EA, 20 min/d for 30 d, 4HZ, continuous waveform (BL23, GV14, GV20)	No treatment	Escape latency	*P* < 0.05
Yang 2014 CG [20]	Wistar rats (12/12)	NR	200–250	2-VO	manual acupuncture, 360 min/d for 21 d (Frontal region, frontoparietal region and parietal region)	No treatment	Escape latency Swimming distance	*P* < 0.05 *P* < 0.05
Yang 2014 CG [20]	Wistar rats (12/12)	NR	200–250	2-VO	manual acupuncture, 360 min/d for 21 d (Frontal region, frontoparietal region and parietal region)	No treatment	Escape latency Swimming distance	*P* < 0.05 *P* < 0.05
Zhang 2014 [21]	Wistar rats (10/10)	NR	300–320	EO	manual acupuncture, 30 s/d for 21 d (CV6, CV12, CV17, SP10, ST36)	Placebo-acupuncture	Escape latency Frequency crossing former platform Duration in former platform position	*P* < 0.01 *P* < 0.01 *P* < 0.01
Li 2015 [22]	Wistar rats (11/11)	NR	320–360	EO	manual acupuncture,30 s/d for 14 d (ST36)	Placebo-acupuncture	Escape latency	*P* < 0.05
Li 2015 [23]	Wistar rats (10/10)	2	300–320	EO	manual acupuncture, 30 s/d for 14 d (ST36	Placebo-acupuncture	Escape latency	*P* < 0.05
Wang 2015 [24]	Wister rats(10/10)	NR	200–220	2-VO	manual acupuncture, 30 s/d for 21 d (CV6, CV12, CV17, SP10, ST36)	Placebo-acupuncture	Escape latency Swimming speed Duration in former platform position	*P* < 0.05 *P* > 0.05 *P* < 0.05
Fang 2016 [25]	SD rats(18/18)	9	300–450	MCAO	EA, 20 min/d for 30 d, 30HZ, 6-15 V, sparse wave (BL23, GV14, GV20)	No treatment	Escape latency	*P* < 0.05
Li 2016 [26]	Wister rats (14/14)	NR	270–320	2-VO	manual acupuncture, 30 s/d for 14 d (ST36)	Placebo-acupuncture	Escape latency Duration in former platform position Swimming speed	*P* < 0.01 *P* < 0.01 *P* > 0.05

*Na* number of animals in the acupuncture group, *EA* electroacupuncture, *Nc* number of animals in the control group, *SD* Sprague Dawley, *4-VO* 4-vessel occlusion, *EO* embolic occlusion, *2-VO* bilateral common carotid artery occlusion, *NR* no record, *PG* positive control group, *CG* cluster-needling group, *MCAO* middle cerebral artery occlusion

### Model preparation method

Various methods were used to establish a vascular dementia (VD) model in the different studies (Table 1) [29]. The 4-vessel occlusion (4-VO) method was used in two studies [11, 13]; 7 studies used bilateral common carotid artery occlusion (2-VO) [15, 17–20, 24, 26]; 6 studies used embolic occlusion (EO) [12, 14, 16, 21–24]; and the remaining trial used middle cerebral artery occlusion (MCAO) [25].

### Description of acupuncture

A range of acupuncture techniques was employed with regard to the combinations of acupoints, the stimulation method (EA or manual acupuncture) and manipulation. Five studies used acupoints CV6 (Zhongji), CV12 (Zhongwan), CV17 (Danzhong), SP10 (Xiehai), ST36 (Zusanli) [12, 14, 16, 21, 24], which was the most commonly used technique. Three studies chose to apply acupuncture treatment to a single acupoint, ST36 (Zusanli) [22, 23, 26]. Four studies used BL23 (Shenshu), GV14 (Dazhui), and GV20 (Baihui) [17–19, 25]. Two studies used GV14 (Dazhui) and GV20 (Baihui) [11, 15]. One study used BL17 (Geshu), BL20 (Pishu), BL23 (Shenshu), and GV20 (Baihui) [13]. The final study had two acupuncture groups [20]: the group called the positive control group used GV14 (Dazhui) and GV20 (Baihui), and the other group, the cluster-needling group, used the frontal region, the frontoparietal region, and the parietal region. Nine studies chose manual acupuncture as the method of stimulation [12, 14, 16, 20–24, 26], while the other seven studies used electroacupuncture [11, 13, 17–19, 25]. Almost all of the studies that used manual acupuncture adopted 30s as their treatment duration; the exception was the study that had two acupuncture groups [20], the positive control group and the cluster-needling group, which used 60 min and 360 min. Of the 7 EA studies, six of the studies adopted continuous waves [11, 13, 15, 17–19], of which the frequency ranged from 4 Hz to 150 Hz, and the current density was from 1 to 2 mA. The remaining study adopted sparse wave [25], of which the frequency was 30 Hz. A summary of the acupuncture treatment protocols is shown in Table 1.

### Control interventions

Ten studies adopted some interventions in the control groups. The Western medicine nimodipine was used as a control intervention in three studies [11, 13, 15], and the remaining seven studies adopted placebo-acupuncture as a control intervention [12, 14, 16, 22–24, 26].

### Study quality assessment

The study quality scores ranged from 4 to 8. Four studies scored points in 4 items [13, 16, 21, 23]; four studies scored points in 5 items [11, 12, 14, 17]; six studies scored points in 6 items [15, 18, 19, 24–26]; one study scored 7 points [22]; and the remaining study scored 8 points [20]. All of the included studies were published in peer-reviewed journals. Eleven of the studies mentioned control of temperature [11–13, 15, 18–20, 22, 24–26], which included room temperature or the temperature of the water in the maze. All of the studies adopted random allocation. Blinded building of the model was adopted in 13 studies [11, 12, 14–17, 19–22, 24–26]. Blinded outcome assessment was adopted in 2 studies [22, 23]. Twelve studies stated the use of anesthetics without marked intrinsic neuroprotective properties [11, 12, 14, 15, 17–20, 22–24, 26]. No study adopted a diabetic, hypertensive or aged animal model, and no study reported sample size calculations. Eight studies mentioned compliance with animal welfare regulations [14, 15, 18, 20, 21, 23–26]. Seven studies stated possible conflicts of interest [13, 16–18, 20, 22, 25]. The study quality assessment is shown in Table 2.

**Table 2:** Methodological quality assessment of the included studies

Study	1	2	3	4	5	6	7	8	9	10	Score
Wang 2004 [11]	Y	Y	Y	Y	N	Y	N	N	N	N	5
Yu 2005 [12]	Y	Y	Y	Y	N	Y	N	N	N	N	5
Shao 2008 [13]	Y	Y	Y	N	N	U	N	N	N	Y	4
Wang 2009 [14]	Y	N	Y	Y	N	Y	N	N	Y	N	5
Wei 2011 [15]	Y	Y	Y	Y	N	Y	N	N	Y	N	6
Zhao 2011 [16]	Y	N	Y	Y	N	U	N	N	N	Y	4
Zhu 2011 [17]	Y	N	Y	Y	N	Y	N	N	N	Y	5
Zhu 2012 [18]	Y	Y	Y	N	N	Y	N	N	Y	Y	6
Zhu 2013 [19]	Y	Y	Y	Y	N	Y	N	N	N	N	6
Yang 2014 [20]	Y	Y	Y	Y	N	Y	N	N	Y	Y	8
Zhang 2014 [21]	Y	N	Y	Y	N	U	N	N	Y	N	4
Li 2015 [22]	Y	Y	Y	Y	Y	Y	N	N	N	Y	7
Li 2015 [23]	Y	N	Y	N	Y	Y	N	N	N	N	4
Wang 2015 [24]	Y	Y	Y	Y	N	Y	N	N	Y	N	6
Fang 2016 [25]	Y	Y	Y	Y	N	U	N	N	Y	Y	6
Li 2016 [26]	Y	Y	Y	Y	N	Y	N	N	Y	N	6

(1) Peer-reviewed journal. (2) Temperature control. (3) Animals were randomly allocated. (4) Blind established model. (5) Blinded outcome assessment. (6) Anesthetics used without marked intrinsic neuroprotective properties. (7) Animal model (diabetic, advanced age or hypertensive). (8) Calculation of sample size. (9) Statement of compliance with animal welfare regulations. (10) Possible conflicts of interest

Y, Yes(low risk bias); N, No(high risk bias); U, Unclear

### Morris water maze outcomes analyses

#### Escape latency

**Fig. 2:**
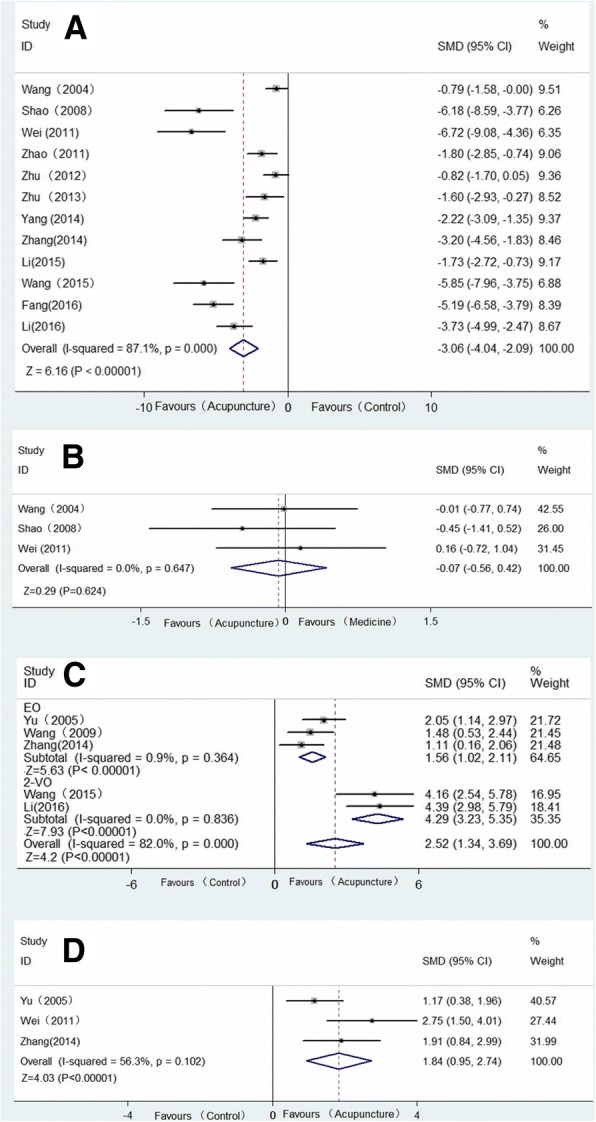
Forest plot showed that escape latency decreases with acupuncture therapies in vascular models. Effect of acupuncture on outcomes of the water maze: effect on (**a**) escape latency time versus the control group; (**b**) escape latency time versus nimodipine; (**c**) duration in original platform; (**d**) frequency of crossing former platform

Twelve studies adopted escape latency as an outcome index. And all of these studies reported the positive effectiveness of acupuncture on reducing escape latency, except for the one study [20] that was designed with two acupuncture groups, the positive control group and cluster-needling group, which showed negative results and positive results, respectively. Of these 12 studies, six of them provided detailed data regarding the marked effect of acupuncture [11, 13, 15, 18–20], and we extracted the data from the other six studies that demonstrated the data in a graphical representation by using GetData software [16, 21–26]. To avoid double-counting [30], the effects of different acupuncture intervention arms included in a single study were averaged and entered once in the analysis [20] [*n* = 280, SMD = − 3.06, 95%CI (− 4.04~ − 2.09), *p* < 0.00001; heterogeneity I^2^ = 87.1%, random effects model, Fig. 2a] [31–33]. Three studies compared the effect of acupuncture with nimodipine [11, 13, 15], and all three of these studies concluded that escape latency was not significantly different between the medication group and the acupuncture group [*n* = 64, SMD = − 0.07, 95% CI (− 0.56 ~ 0.42), *p* = 0.775 > 0.05; heterogeneity I^2^ = 0%, fixed effect model, Fig. 2b].

#### Duration in original platform

Five studies showed the effect of acupuncture on improving the duration in the quadrant of the former platform compared with the control group (VD group or placebo-acupuncture group) [12, 14, 21, 24, 26]. All five of these studies reported positive results, but none of them provided detailed data [13]; we therefore extracted the data from the figures [*n* = 119, SMD = 2.52, 95%CI (1.34 ~ 3.69), *p* < 0.00001; heterogeneity I^2^ = 82.0%, random effects model, Fig. 2c].

#### Frequency of crossing former platform

Three studies reported the positive results of acupuncture on increasing the frequency of crossing the former platform location [12, 15, 21] [*n* = 69, SMD = 1.84, 95% CI (0.95 ~ 2.74), *p* < 0.00001; heterogeneity I^2^ = 56.3%, random effects model, Fig. 2d].

There was no significant difference observed in the animals’ swimming speed in the water maze in the two studies that measured it, although the detailed data were unavailable [24, 26].

### Subgroup analyses

#### Escape latency

##### Animal species

Acupuncture was found to have a remarkable effect on reducing escape latency time in both Sprague-Dawley rats (SMD –3.33, 95% CI –5.18 ~ − 1.47; *p* < 0.00001) and Wister rats (SMD –2.85, 95% CI –3.79~ − 1.91; *p* = 0.002). The subgroup analysis observed that Wister rats had slightly reduced heterogeneity (I^2^ = 73.5%), while Sprague-Dawley rats did not (I^2^ = 92.1%).

##### Acupuncture methods

We performed a subgroup analysis of the methods of acupuncture used (Table 3) and observed significant effects of manual acupuncture (SMD –3.11, 95% CI –4.23 ~ − 2.00; *p* = 0.002) and electro-acupuncture (SMD –3.05, 95% CI –4.56~ − 0.78; *p* < 0.00001) on reducing escape latency time. Subgroup analysis of the acupuncture methods indicated that manual acupuncture had reduced heterogeneity (I^2^ = 75.9%), while electroacupuncture did not (I^2^ = 90.5%).

**Table 3:** Subgroup analysis for the effect of acupuncture on reducing escape latency time

		SMD	LL	HL	Degrees of freedom	Heterogeneity	Effect size	
						I^2^	Z	P
Species	SR	−3.33	−5.18	−1.47	5	92.10%	3.52	*P* < 0.00001
	WR	−2.85	−3.79	−1.91	5	73.50%	5.93	*P* = 0.002
Modeling	2-VO	−2.97	−4.21	− 1.73	6	85.80%	4.69	*P* < 0.00001
	4-VO	−3.36	−8.63	1.92	1	94.30%	1.25	*P* = 0.212
	EO	−2.38	−3.82	−0.95	1	65.80%	3.26	*P* = 0.001
	MCAO	−5.19	−6.58	−3.79	0	–	7.29	*P* < 0.00001
Methods	EA	−3.05	−4.56	−1.53	6	90.50%	3.94	*P* < 0.00001
	MA	−3.11	−4.23	−2.00	4	75.90%	5.47	*P* = 0.002
OVERALL		−3.06	−4.04	−2.09	11	87.10%	6.04	*P* < 0.00001

*SR* Sprague–Dawley Rats, *WR* Wister Rats, *2-VO* bilateral common carotid artery occlusion, *VO* 4-vessel occlusion, *EO* embolic occlusion, *MCAO* middle cerebral artery occlusion, *EA* Electroacupuncture, *MA* Manual acupuncture

##### Modeling methods

We also conducted a subgroup analysis of the modeling methods (Table 3) [31]. This subgroup analysis showed that acupuncture had a significant effect on 2-VO models (SMD –2.97, 95% CI –4.21~ − 1.73; *p* < 0.00001), EO models (SMD –2.38, 95% CI –3.82~ − 0.95; *p* = 0.001), and MCAO models (SMD –5.29, 95% CI –6.58~ − 3.79; *p* < 0.00001), but no remarkable difference was found in the 4-VO models (SMD –3.36, 95% CI –8.63 ~ 1.92; *p* = 0.212 > 0.05).

#### Duration in original platform

##### Modeling methods

For all five of the studies that regarded duration in original platform as an outcome measure, adopted electroacupuncture, and used Wister rats, we only conducted a subgroup analysis of the modeling methods (Fig. 2c). Both the EO models (SMD 1.56, 95% CI 1.02 ~ 2.11; *p* < 0.00001) and the 2-VO models (SMD 4.92, 95% CI 3.23 ~ 5.35; *p* < 0.00001) found a remarkable effect with the use of electroacupuncture, and this subgroup analysis significantly reduced the heterogeneity (EO: I^2^ = 0.9%, 2-VO: I^2^ = 0%).

### Publication bias test

**Fig. 3:**
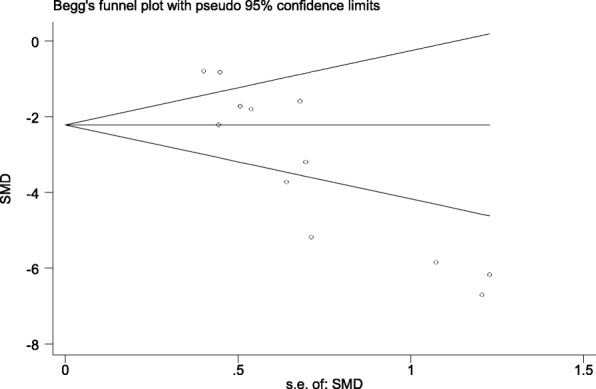
Begg’s test for the outcome of escape latency

We performed a publication bias test for the outcome of escape latency using Egger’s test (Pr > |z| = 0.001 < 0.05, continuity corrected) and Begg’s test (Pr > |z| = 0.005 < 0.05, continuity corrected, Fig. 3). The results of these two tests indicated the potential existence of publication bias across all of the included studies [34]. We did not conduct a publication bias test for the other outcome measures, because there were fewer than ten included studies for each measure [35].

### Signaling pathways

Of the sixteen included studies, fifteen described possible mechanisms of acupuncture in ameliorating cognitive function. The main signaling pathways were summarized in three aspects, oxidative stress damage reduction, nerve apoptosis suppression and neurogenesis. See Table 4 [36, 37].

**Table 4:** Proposed mechanisms

Study	Findings & Proposed mechanisms
Wang 2004 [11]	• Reduced NO, NOS and MDA • Increased SOD and GSH-Px
Shao 2008 [13]	Increased AVP and SS
Wang 2009 [14]	• Up-regulating the expression of Bcl-2 • Counter-regulated the pro-apoptotic Bax
Wei 2011 [15]	Promoting synaptic function and structure
Zhao 2011 [16]	Enhanced hexokinase, pyruvate kinase and glucose 6 phosphate dehydrogenase activities
Zhu 2011 [17]	Inhibiting expression of p53 and Noxa
Zhu 2012 [18]	Increased p70S6K and ribosomal protein S6
Zhu 2013 [19]	Increased mTOR and eIF4E
Yang 2014 [20]	Increased hippocampal ACh, DA, and 5-HT
Zhang 2014 [21]	Increased CBF
Li 2015 [22]	Increase pyramidal neuron number in hippocampal CA1 area
Li 2015 [23]	• Inhibited PDE activity • Activated ERK and cAMP/PKA/CREB
Wang 2015 [24]	Enhanced Nrf2
Fang 2016 [25]	Decreased TNF-α mRNA, IL-6 mRNA and IL-1β mRNA
Li 2016 [26]	• Increased complex I, II, IV and cox IV • Decreased ROS

*NO* nitric oxide, *NOS* nitric oxide synthase, *MDA* malondialdehyde, *GSH-Px* glutathione peroxidase, *AVP* arginine vasopressin, *SS* somatostatin, *Bcl-2* B-cell lymphoma-2, *Bax* Bcl-2 associated X protein, *P53* Tumor protein P53, *P70S6K* P70 ribosomal protein S6 kinase, *mTOR* mammalian target of rapamycin, *eIF4E* eukaryotic translation initiation factor, *Ach* acetylcholine, *DA* dopamine, *5-HT* 5-hydroxytryptamine, *CBF* cerebral blood flow, *PDE* phosphodiesterase, *ERK* extracellular signal-regulated kinase, *cAMP* 3′,5′-cyclic AMP/protein kinaseA, *PKA* protein kinaseA, *CREB* cAMP/PKA/cAMP response element binding protein, *Nrf2* nuclear factor erythroid-related factor 2, *TNF* tumor necrosis factor, *IL* interleukin, *coxIV* cytochrome oxidase IV, *ROS* reactive oxygen species

## Discussion

To the best of our knowledge, this report describes the first systematic meta-analysis exploring the effect of acupuncture on vascular dementia in animal experiments with the results of the Morris water maze test as the outcome assessment.

### Implications

This study indicates that electroacupuncture could increase the duration in the former platform quadrant both in EO models (SMD = 1.56, 95% CI: 1.02 ~ 2.11; *p* < 0.00001; heterogeneity: I-squared = 0.9%) and 2-VO models (SMD = 4.29, 95% CI: 3.23 ~ 5.35; *p* < 0.00001; heterogeneity: I-squared = 0%) compared with the control groups. This finding suggests that acupuncture may play a potential role in ameliorating cognitive dysfunction in animal models. A previous study indicates that the addition of acupuncture therapy to routine care may have beneficial effects on improvements in cognitive status but limited efficacy on health-related quality of life in vascular dementia patients [38].

The high heterogeneity between the studies based on escape latency time cannot be completely explained. We conducted subgroup analyses based on the method of stimulation, the modeling method and the animal species used, but no evident cause was found. Meanwhile, the subgroup analysis of the modeling methods that regarded the duration in former platform as an outcome measure obviously reduced the heterogeneity. We found all five of the studies in this analysis adopted the same method of stimulation, the same animal species, and similar acupoint combinations. Four of the five studies used CV6, CV12, CV17, SP10, and ST36, the other study used ST36. These findings indicate that, in addition to the differences in stimulation method, modeling methods and animal species used, acupoint combinations may be another source of heterogeneity among the studies.

Systematic reviews can help promote the methodological quality of preclinical animal studies. Systematic reviews can help promote the methodological quality of preclinical animal studies. Essential methodological details are significant to measure the quality of a body of evidence and to assess the bias risk in animal trials. However, the insufficiency of the methodology is evident in many aspects of the present study. For instance, an adequate target animal model represents an important aspect to improve the quality of the experimental design. All included studies were performed on healthy and young animals. However, vascular dementia [39] generally occurs in aged, diabetic or hypertensive patients; therefore, experiments using young and healthy animals may overestimate the effectiveness of the intervention [10]. Hence, appropriate target animal models (hypertensive, advanced age and diabetic) should be used in future experimental research.

The included studies investigated several signaling pathways to gain a better understanding of the mechanism of improving cognitive function via acupuncture, including modulating the production and degradation of free radicals to reduce brain damage [11], exerting anti-apoptotic effects by up-regulating B-cell lymphoma-2 (Bcl-2) and counter-regulating Bcl-2-associated X protein (Bax) to protect neurons [14], enhancing glucose metabolism in the brain [16], reducing tumor protein P53 and Noxa expression to protect pyramidal cells from apoptosis [17], increasing cerebral blood flow for increased glucose and oxygen supply to neurons [21], exerting neuroprotective effects via an antioxidative pathway mediated by nuclear factor erythroid2-related factor 2 (Nrf2) [24], and increasing complex enzymes to protect neurons from oxidative stress [26]. The present study indicated that acupuncture protects neurons during vascular dementia mainly through enhancing oxygen and glucose metabolism and anti-apoptosis and antioxidant properties. However, the signaling pathways targeted by acupuncture were infrequently and incompletely reported. Therefore, this field should be further explored in future clinical studies.

### Limitations

This systematic meta-analysis has several limitations, the first of which is language bias. We limited the language of our searches to English only, which may cause potential publication bias. The results may have differed if we had included studies reported in Chinese, Korean, Japanese or other languages. Second, the total sample size was still not big enough, although we made a concerted effort to search all of the studies that met the inclusion criteria. Third, the quality of the included studies was unsatisfactory, which had an important influence on the results of the systematic meta-analysis. Fourth, evident availability bias was caused by two included studies from which the data cannot be extracted [35]. We tried to contact the authors by e-mail, but we received no reply. Fifth, we performed Begg’s test and Egger’s test for publication bias assessment, and the results indicated potential publication bias.

In view of the limitations above, we recommend that non-English language literature should be included in future systematic meta-analyses. Furthermore, blind outcome assessments, use of target animal models (hypertensive, advanced age and diabetic), calculation of sample size, statement of compliance with animal welfare regulations, possible conflicts of interest and detailed data publishing should be considered in future animal model studies.

## Conclusions

From a methodological perspective, animal experiments should be standardly and appropriately designed and transparently reported. Acupuncture may play a potential role in ameliorating cognitive dysfunction in animal models. Furthermore, our findings indicates that acupuncture could protect neurons in animal models of vascular dementia through enhanced oxygen and glucose metabolism, as well as antioxidant and anti-apoptosis effects. Thus, this field should be further explored in vascular dementia clinical trials.

## Additional files

Additional file 1: PRISMA 2009 Checklist. (DOC 57 kb)

Additional file 2: Search Strategies. (DOCX 12 kb)

## Abbreviations

2-VO: Bilateral common carotid artery occlusion
4-VO: 4-vessel occlusion
CI: Confidence interval
EA: Electroacupuncture
EO: Embolic occlusion
MCAO: Middle cerebral artery occlusion
SD: Sprague–Dawley
SMD: Standard mean differences
VD: Vascular dementia
