# Supplementary material for: Effects of acupuncture on vascular dementia (VD) animal models: a systematic review and meta-analysis
Source: BMC Complement Altern Med. 2018 Nov 13;18:302. doi: 10.1186/s12906-018-2345-z (PMC6234685; doi:10.1186/s12906-018-2345-z)
Supplement: Supplementary file 2 — Search Strategies. (DOCX 12 kb) [file 12906_2018_2345_MOESM2_ESM.docx]

**PubMed**

(("dementia, multi-infarct"[MeSH Terms] OR ("dementia"[All Fields] AND "multi-infarct"[All Fields]) OR "multi-infarct dementia"[All Fields] OR ("multi"[All Fields] AND "infarct"[All Fields] AND "dementia"[All Fields]) OR "multi infarct dementia"[All Fields]) OR ("dementia, vascular"[MeSH Terms] OR ("dementia"[All Fields] AND "vascular"[All Fields]) OR "vascular dementia"[All Fields] OR ("vascular"[All Fields] AND "dementia"[All Fields]))) AND ((("acupuncture"[MeSH Terms] OR "acupuncture"[All Fields] OR "acupuncture therapy"[MeSH Terms] OR ("acupuncture"[All Fields] AND "therapy"[All Fields]) OR "acupuncture therapy"[All Fields]) OR ("electroacupuncture"[MeSH Terms] OR "electroacupuncture"[All Fields])) OR ("acupuncture points"[MeSH Terms] OR ("acupuncture"[All Fields] AND "points"[All Fields]) OR "acupuncture points"[All Fields] OR "acupoint"[All Fields])) AND English[lang]

**Embase**

#1 'acupuncture'/exp OR 'acupuncture'

#2 'electroacupuncture'/exp OR 'electroacupuncture'

#3 'acupoint'/exp OR 'acupoint'

#4 'multi infarct' AND ('dementia'/exp OR dementia)

#5 'multiinfarct dementia'/exp OR 'multiinfarct dementia'

#6 vascular AND ('dementia'/exp OR dementia)

#7 #1 OR #2 OR #3

#8 #4 OR #5 OR #6

#9 #7 AND #8 AND [english]/lim

**Ovid**

1 Acupuncture Therapy/

2 Electroacupuncture/

3 Acupuncture Points/ or Acupuncture/

4 acupoint*.mp. [mp=title, abstract, original title, name of substance word, subject heading word, keyword heading word, protocol supplementary concept word, rare disease supplementary concept word, unique identifier, synonyms]

5 acupuncture.mp. [mp=title, abstract, original title, name of substance word, subject heading word, keyword heading word, protocol supplementary concept word, rare disease supplementary concept word, unique identifier, synonyms]

6 electroacupuncture.mp. [mp=title, abstract, original title, name of substance word, subject heading word, keyword heading word, protocol supplementary concept word, rare disease supplementary concept word, unique identifier, synonyms]

7 Dementia, Vascular/

8 Dementia, Multi-Infarct/

9 vascular dementia.mp. [mp=title, abstract, original title, name of substance word, subject heading word, keyword heading word, protocol supplementary concept word, rare disease supplementary concept word, unique identifier, synonyms]

10 multi-infarct dementia.mp. [mp=title, abstract, original title, name of substance word, subject heading word, keyword heading word, protocol supplementary concept word, rare disease supplementary concept word, unique identifier, synonyms]

11 multiinfarct dementia.mp. [mp=title, abstract, original title, name of substance word, subject heading word, keyword heading word, protocol supplementary concept word, rare disease supplementary concept word, unique identifier, synonyms]

12 1 or 2 or 3 or 4 or 5 or 6

13 7 or 8 or 9 or 10 or 11

14 12 and 13

15 limit 14 to english language
